# Coronavirus Disease in Children: A Single-Center Study from Western Saudi Arabia

**DOI:** 10.1155/2021/9918056

**Published:** 2021-07-27

**Authors:** Rafat M. Mosalli, Sumayyah Ahmed Nezar Kobeisy, Nawaf M. Al-Dajani, May Abu Ateeg, Mostafa A. Ahmed, Wael M. Meer, Hussain Y. Al-Saeedi, Saleh A. Al-Harbi

**Affiliations:** ^1^Department of Pediatrics, Umm Al Qura University, Makkah, Saudi Arabia; ^2^Department of Pediatrics, International Medical Center, Jeddah, Saudi Arabia; ^3^Department of Pediatrics, Doctor Soliman Fakeeh Hospital, Jeddah, Saudi Arabia; ^4^Department of Pediatrics, King Abdulaziz University, Jeddah, Saudi Arabia

## Abstract

**Introduction:**

Local data in Saudi Arabia regarding pediatric SARS-CoV-2 infection is limited. This study is aimed at adding insight regarding the effect of the novel coronavirus on pediatric patients by studying the presentation, laboratory parameters, and disposition of SARS-CoV-2-infected pediatric patients in one center in Jeddah, Saudi Arabia. *Methodology*. A retrospective study was conducted at the International Medical Center (IMC) in Jeddah, Saudi Arabia, to assess features of pediatric patients admitted with COVID-19 from April 2020 to September 2020.

**Results:**

A total of 43 patients were found to meet the study inclusion criteria. The most common presenting symptom was fever (53.5%) in study participants followed by complaints of cough, runny nose, and shortness of breath (37.2%). Lymphocytopenia was evident among 60% of those studied. Elevated C-Reactive Protein was remarkable in 24.9%. More than half of those (53.5%) studied required only supportive treatment.

**Conclusion:**

COVID-19 disease for the most part is mild in children with a varying clinical picture and nonspecific laboratory parameters. Further, large-scale national-based studies are needed to help in the early identification of pediatric cases at risk of complication due to COVID-19 infection hence providing proper and timely management, identifying population-specific disease pattern and perhaps targeted immunization.

## 1. Introduction

The highly contagious novel coronavirus outbreak was first reported to the Chinese authorities on December 31, 2019 [1–2]. As a worldwide pandemic ensued, as of March 2021, more than 295,000 cases of COVID-19 have been reported in Saudi Arabia with more than 6,000 deaths [3]. Clinical characteristics have been commonly reported as fever and respiratory symptoms and have ranged to include renal injury, central nervous system involvement, and gastrointestinal complications [4]. In the pediatric age group, the rate of morbidity and mortality associated with COVID-19 is not as severe as in adults [5]. Preemptive management has been used through artificial intelligence models which anticipate the number of people infected with COVID-19 several days in advance in hotspot regions not just across Europe the Americas but in the Middle East as well and specifically in Saudi Arabia [6–8]. This may lead to help health care professionals to prepare hospital settings, facilities, and personal protective equipment as well as increasing public awareness [6, 8]. Local data in Saudi Arabia regarding pediatric SARS-CoV-2 infection is limited [9–12]. This study is aimed at adding insight regarding the effect of the novel coronavirus on pediatric patients by studying the presentation, laboratory parameters, and disposition of SARS-CoV-2-infected pediatric patients in one center in Jeddah, Saudi Arabia.

## 2. Materials and Methods

A retrospective study was conducted at the International Medical Center (IMC) in Jeddah, Saudi Arabia, to assess features of pediatric patients admitted with COVID-19 from April 2020 to September 2020. Collection of medical records for analysis was approved by the International Medical Center Institutional Review Board (IMC IRB), and the guidelines outlined in the Declaration of Helsinki were followed. Patient health records were extracted from the hospital information system. Those included in the study were children less than 14 years of age with positive nasopharyngeal RT-PCR for SARS-CoV-2 and requiring hospital admission. Files were evaluated for demographic features, clinical presentation, laboratory parameters, radiological findings, and treatment outcome.

## 3. Results

### 3.1. Demographic Features

A total of 43 patients were found to meet the study inclusion criteria. A little more than half (53.5%) were female, and almost three-quarters (76.7%) were ethnic to Saudi Arabia. The great majority (72.1%) presented in April and May. Almost one-third of those included in the study were toddler age (37.2%) followed by 34.9% between 5 and 10 years of age. Detailed demographic data can be found in Table 1.

### 3.2. Clinical Features

The most common presenting symptom was fever (53.5%) in study participants followed by complaints of cough, runny nose, and shortness of breath (37.2%). There were 27.9% that also presented without symptoms, and almost three-fourths (74.4%) had household contact of COVID-19. Clinical examination in hospital revealed that the average pulse oxygen saturation of patients was 97.5% and the most common sign was fever in 39.5% followed by pharyngitis in 20.9%. However, 53.5% had unremarkable physical exam findings. On radiological evaluation, 41/43 (95.4%) had insignificant chest X-ray findings. Comprehensive data regarding patient signs and symptoms can be found in Table 2.

### 3.3. Laboratory Features

Among those studied, only 40 underwent laboratory testing. Lymphocytopenia was evident among 60% of those studied. Elevated C-Reactive Protein was remarkable in 24.9%. Renal function as measured by the creatinine level was elevated in almost one third (30.3%), while alanine transaminase was abnormal in 26.1%.  Table 3 demonstrates detailed laboratory findings.

### 3.4. Management and Outcome

More than half of those (53.5%) studied required only supportive treatment which included cardiopulmonary monitoring, antipyretics, and supplemental oxygen via a nasal cannula at a rate of 2-4 L/min for a saturation < 92%. 37.2% required additional antibiotic therapy for secondary bacterial infection, and only one patient received the antiviral medication, Oseltamivir, due to coinfection with influenza A. One patient studied was admitted to the intensive care unit, required additional management with dexamethasone, IVIG, tocilizumab, granulocyte colony stimulating factor (G-CSF), platelet and blood transfusions, and invasive respiratory support. While this patient was the only mortality registered among those studied, however, it is worth mentioning this child suffered from Chediak-Higashi syndrome and presented with a picture of multisystem inflammatory syndrome in children (MIS-C). Patient management can be seen in Table 4.

## 4. Discussion

The novel coronavirus affects males and females equally as well as all pediatric age groups; however, it seems that the younger age groups are more symptomatic [13]. The results of this study show a bimodal age presentation of the patients that presented to the International Medical Center, mainly between 1-3 years and 5-10 years of age. This could be due to the limited sample size or a true reflection of COVID-19 affliction in the pediatric population in Saudi Arabia.

The most common clinical symptoms in pediatrics have been reported by Cui et al. as fever, cough, and shortness of breath and gastrointestinal symptoms as vomiting and diarrhea and fatigue [14]. Atypical presentations of COVID-19 disease in the literature were frequently documented in neonates or in children with underlying medical conditions such as asthma, heart disease or immunosuppression [15]. More than half of the patients that were included in this study did not have clinically remarkable findings on clinical examination, and many were asymptomatic on presentation similar to patients included in one systematic review where 19.3% of patients were asymptomatic as well [16]. There has been conflicting evidence which has shown children to have higher rates of symptomatology associated with SARS-CoV-2 infection [17–18]. A study conducted in the central region of Saudi Arabia showed that the rate of severe SARS-CoV-2 associated with MIS-C was 5/88 (5.7%) [12] whereas the percentage of MIS-C at the IMC which is in the western region was approximately 2.3% (1/43). This demonstrates that within the country of Saudi Arabia there are regional differences in severity rates. The rate of SARS-CoV-2 exposure or household contact in this study was similar to that in other studies indicating high infectivity [15]. However, it has been concluded by Bergrath et al. that symptoms of COVID-19 may be misleading in critically ill patients and COVID-19 pharyngeal swabs may also show false negative results in these patients [19].

Contrary to the findings of this study, lymphocytosis and leukocytosis were found to be evident among children with COVID-19 [16]. Serum inflammatory markers such as CRP were elevated in several studies [11, 13, 16]. Locally in Saudi Arabia, the study conducted by Al Harbi et al. in the Western region found eosinopenia to be the most prevalent among their patient population. The discrepancies between laboratory findings may be attributed to the inflammatory state of each patient and are nonspecific parameters for COVID-19.

Supportive management for children diagnosed with the novel coronavirus is the main stream approach [20]. The antiviral medication, Remdesivir, impairs viral replication; however, there is limited data on children [20]. Of the 43 patients in this study, only one patient developed severe disease and required ICU admission and aggressive therapy and unfortunately did not survive. This patient had an immunosuppressive illness which supports that the novel coronavirus is aggressive in children with underlying medical conditions. This study has several limitations which include the small sample size and being restrained to one center in Jeddah, Saudi Arabia. Although weather across Saudi Arabia is usually consistent, they may vary due to different altitudes and humidity levels. Some studies have shown that there may be a correlation on the spread of the novel corona virus and the spread of the disease [21–22]. However, the findings presented here do emphasize the wide clinical presentation of the novel coronavirus in children, and further studies are needed in various regions of Saudi Arabia to determine whether local factors in each area play a role.

## 5. Conclusion

COVID-19 disease for the most part is mild in children with a varying clinical picture and nonspecific laboratory parameters. Further, large-scale prospective multicenter national and international middle eastern-based studies are needed to better understand the epidemiology and help in the early identification of pediatric cases at risk of complication due to COVID-19 infection hence providing proper and timely intervention, identifying population-specific disease pattern and perhaps targeted immunization.

## Figures and Tables

**Table 1:** Demographic data.

	Total COVID-19 cases, *n* (%)
Sex	*n* = 43 (%)
Male	20 (46.5)
Female	23 (53.5)
Nationality	*n* = 43 (%)
Saudi	33 (76.7)
Other	10 (23.3)
Length of stay (days)	*n* = 43
Mean ± SD	3.7 ± 2.1
Minimum–maximum	1–9
Month of presentation	*n* = 43 (%)
April	10 (23.3)
May	21 (48.8)
June	4 (9.3)
July	4 (9.3)
August	1 (2.3)
September	3 (7.0)
Age	*n* = 43
Mean ± SD	5.3 ± 4.3
Minimum–maximum	0–13
Age group	*n* = 43 (%)
<1 year	4 (9.3)
1-3 years	16 (37.2)
>3-5 years	2 (4.7)
>5-10 years	15 (34.9)
>10 years	6 (14.0)

**Table 2:** Signs and symptoms.

	Total COVID-19 cases, *n* (%)
Pulse oxygen saturation	*n* = 43
Mean ± SD	97.5 ± 0.83
Minimum–maximum	96–99
Presenting symptoms	*n* = 43 (%)
Fever	23 (53.5)
Upper respiratory tract symptoms	16 (37.2)
Asthma exacerbation	1 (2.3)
Central nervous system symptoms	2 (4.7)
Rash	1 (2.3)
Lymphadenitis	1 (2.3)
Gastrointestinal symptoms	12 (27.9)
Asymptomatic	12 (27.9)
History of COVID-19 contact	*n* = 43 (%)
Household	32 (74.4)
Unknown	11 (25.6)
Clinical signs	*n* = 43 (%)
Fever	17 (39.5)
Mild dehydration	7 (16.3)
Tonsillitis	2 (4.7)
Otitis media	1 (2.3)
Pharyngitis	9 (20.9)
Cough/sneezing	1 (2.3)
Loss of taste/smell	1 (2.3)
Cervical lymphadenitis	1 (2.3)
Myalgia	1 (2.3)
Rash	1 (2.3)
Unremarkable	23 (53.5)
Chest X-ray	*n* = 43 (%)
Bronchopneumonia	1 (2.3)
Increased broncho-vascular markings	1 (2.3)
Unremarkable	41 (95.4)

**Table 3:** Lab parameters.

Lab parameter (*n*)	Minimum–maximum	Mean ± SD	Abnormal result, *n* (%)
WBC (40) (10^3^/*μ*L)	3.00-25.60	7.53 ± 3.91	Leukocytosis, 1 (2.5) Leucopenia, 12 (30.0)
ANC (40) (10^3^/*μ*L)	0.27-11.0	2.94 ± 2.16	Neutrophilia, 2 (5.0) Neutropenia, 4 (10.0)
Lymphocyte (40) (10^3^/*μ*L)	0.33-10.50	3.84 ± 2.50	Lymphocytopenia, 24 (60.0)
Eosinophil (40) (10^3^/*μ*L)	0.0-3.30	0.35 ± 0.69	Eosinophilia, 4 (10.0)
Basophil (40) (10^3^/*μ*L)	0.00-0.30	0.04 ± 0.06	Basophilia, 3 (7.5)
Hemoglobin (40) (g/dL)	7.80-17.20	12.49 ± 1.69	Low hemoglobin, 6 (15.0)
Platelets (40) (10^3^/*μ*L)	19-759	302.4 ± 131.4	Thrombocytopenia, 2 (5.0) Thrombocytosis, 7 (7.5)
CRP (37) (mg/L)	0.0-296	22.1 ± 61.2	Elevated CRP, 9 (24.3)
BUN (34) (mg/dL)	0.34-20	4.4 ± 0.6	Elevated BUN, 9.3 (11.8)
Creatinine (33) (mg/dL)	0.17-47	8.1 ± 14.3	Elevated creatinine, 10 (30.3)
Serum Sodium (38) (mmol/L)	134-143	139.3 ± 2.1	Hyponatremia, 1 (2.6)
Serum Potassium (38) (mmol/L)	2.9-6.2	4.5 ± 0.6	Hypokalemia, 2 (5.9) Hyperkalemia, 2 (5.9)
Serum Calcium (10) (mmol/L)	2.2-2.6	2.4 ± 1.7	Hypercalcemia, 2 (20.0)
Serum Phosphate (10) (mmol/L)	1.3-2.0	1.7 ± 0.2	Hyperphosphatemia, 9 (90.0)
ALT (23) (U/L)	8-184	28.8 ± 20.2	Elevated ALT, 6 (26.1)
AST (22) (U/L)	10.2-106	28.8 ± 20.2	Elevated AST, 2 (9.1)

**Table 4:** Patient management.

	Total COVID-19 cases, *n* (%)
Treatment	*n* = 43 (%)
Antibiotic (secondary bacterial infection)	16 (37.2)
Antiviral (Oseltamivir)	1 (2.3)
ICU	1 (2.3)
Dexamethasone	1 (2.3)
Mechanical ventilation/high frequency	1 (2.3)
Outcome	*n* = 43 (%)
Death	1 (2.3)

## Data Availability

Data supporting the findings of this study are available upon request from the corresponding author.
